# Delayed Diagnosis and Missed Opportunities for Early Autism Identification in Brazil: An Exploratory Scoping Review

**DOI:** 10.3390/children13081082

**Published:** 2026-08-15

**Authors:** Bianca Teeny Sallum, Monaliza Ehlke Ozorio Haddad, Maria Gabriela Custódio de Figueiredo, Tiago S. Bara, Vanessa Furlin, Mara L. Cordeiro

**Affiliations:** 1 Ciencias Sociales y Juridicas, Universidad de Salamanca, 37008 Salamanca, Spain; idu054400@usal.es; 2 Instituto de Pesquisa Pelé Pequeno Príncipe, Curitiba 80250-060, PR, Brazil; gabi.figueiredo@mail.utoronto.ca (M.G.C.d.F.); tiago.bara@pelepequenoprincipe.org.br (T.S.B.); vanessa.furlin@pelepequenoprincipe.org.br (V.F.); 3 Instituto Aprendizagem e Desenvolvimento (IAD), Araucária 83702-080, PR, Brazil; monalizahaddad@iadpr.com.br; 4 Faculdades Pequeno Príncipe, Curitiba 80230-020, PR, Brazil; 5 Department of Psychiatry and Biobehavioral Sciences, David Geffen School of Medicine, University of California, Los Angeles, CA 90095, USA

**Keywords:** autism spectrum disorder, diagnostic delay, early diagnosis, developmental surveillance, primary care, autism screening, health system barriers, Brazil

## Abstract

**Highlights:**

**What are the main findings?**

• Delayed autism diagnosis in Brazil reflects multilevel barriers spanning families, providers, referral pathways, and health system organization.
• Primary care played a limited role in early identification, while specialist-centered pathways and regional inequalities contributed to prolonged diagnostic trajectories.

**What are the implications of the main findings?**

• Earlier diagnosis may be improved by strengthening developmental surveillance, referral coordination, and primary health care capacity.
• Future Brazilian research should prioritize standardized reporting, implementation studies, and broader geographic and equity-focused representation.

**Abstract:**

**Background/Objectives**: Timely identification of autism spectrum disorder (ASD) is essential for access to early intervention; however, diagnostic delay remains a persistent challenge. **Methods**: This scoping review synthesized evidence from eight studies (>24,000 participants), most conducted in the Southeast region, on diagnostic pathways, screening practices, and barriers to ASD identification in Brazil, following PRISMA-ScR guidelines. **Results**: Across studies, mean age at diagnosis frequently exceeded 48–60 months, while the interval between first caregiver concern and diagnosis ranged from 24 to 36 months, with tertiary-care samples reporting diagnostic ages approaching 79 months. Diagnosis was predominantly specialist-driven, with limited involvement of primary care providers (PCPs). Later identification was associated with reliance on the public health system in a multinational analysis that included Brazil, while geographic concentration of specialized services and socioeconomic inequalities were identified as reported barriers across the included studies. Race/ethnicity was rarely reported. Delays emerged from interacting multilevel barriers, including limited caregiver awareness, dismissal of parental concerns, inconsistent developmental surveillance, fragmented referral pathways, and shortages of specialists. Structural inequities, particularly geographic disparities and urban concentration of services, compounded these challenges. **Conclusions**: The evidence suggests delayed ASD diagnosis in Brazil reflects systemic gaps in care organization rather than isolated clinical factors. Strengthening PCP-based developmental surveillance and improving referral coordination are key strategies to reduce preventable delays and promote earlier access to intervention.

## 1. Introduction

Timely identification of autism spectrum disorder (ASD) remains a major global public health challenge. ASD is a neurodevelopmental condition characterized by persistent difficulties in social communication and interaction, alongside restricted and repetitive patterns of behavior or interests, according to the *Diagnostic and Statistical Manual of Mental Disorders, Fifth Edition, Text Revision* (DSM-5-TR) [1]. Screening tools such as the Modified Checklist for Autism in Toddlers (M-CHAT) may support early identification, but diagnosis ultimately depends on comprehensive clinical evaluation. Clinical presentation is heterogeneous, which may complicate early recognition within routine developmental surveillance systems.

There is no global consensus on what constitutes a “late” ASD diagnosis. Definitions vary across studies and health system contexts, and although pooled international estimates suggest mean age at diagnosis commonly ranges from approximately 52 to 60 months, the interval between first caregiver concern and confirmed diagnosis often remains prolonged [2,3,4]. Distinguishing age at diagnosis from diagnostic delay is therefore essential, as reductions in mean age do not necessarily reflect shorter intervals between first concern and diagnostic confirmation. Throughout this review, “early identification” refers broadly to the process of recognizing children with possible ASD and facilitating timely referral and diagnostic evaluation, whereas “diagnostic delay” refers specifically to the interval until formal diagnostic confirmation.

Delayed diagnosis has important developmental and social consequences. Earlier identification facilitates timely access to intervention during periods of rapid neurodevelopment, educational accommodations, and family support, whereas prolonged diagnostic trajectories may delay access to appropriate services and exacerbate inequities in care [5,6]. International reviews have identified multilevel determinants of diagnostic timing, including symptom severity, parental advocacy, socioeconomic status (SES), educational attainment, and health system characteristics [7,8]. Evidence also suggests that sex- and gender-related differences in presentation may affect recognition, while socioeconomic and racial inequities may influence access to specialized assessment.

Brazil provides a distinctive context for examining these issues. As a middle-income country with constitutionally guaranteed universal health coverage through the Unified Health System [Sistema Único de Saúde (SUS)], Brazil formally provides nationwide access to primary health care (PHC), developmental monitoring, and referral to specialized services [9]. However, persistent regional and socioeconomic inequalities shape the distribution of PHC infrastructure and specialist services, which remain concentrated in the South and Southeast [5,10,11,12]. Although international comparative analyses have not found statistically significant differences in mean age at diagnosis between high-income and low- and middle-income countries overall, such comparisons may obscure substantial within-country disparities in a large and geographically diverse setting such as Brazil [2].

Existing Brazilian reviews have addressed family burden after diagnosis and challenges related to the translation, validation, and implementation of diagnostic tools [12,13]. However, no prior review has simultaneously mapped the available evidence on age at diagnosis, diagnostic delay, screening practices, referral pathways, and multilevel barriers within the Brazilian context. The available literature is also methodologically heterogeneous, including cross-sectional surveys, administrative datasets, qualitative studies, and small intervention studies, supporting the use of an exploratory scoping review to map the current evidence base and identify knowledge gaps.

Accordingly, this review addressed three questions: (1) What evidence is available in Brazil regarding early ASD identification, screening practices, diagnostic pathways, and barriers to timely diagnosis? (2) Which tools, settings, populations, and outcomes have been studied, and how are “diagnostic delay” and “missed opportunities” defined across studies? (3) How do regional distribution, socioeconomic status, race/ethnicity, and gender relate to reported patterns of diagnostic timing? By mapping the available evidence, this exploratory scoping review aimed to characterize the current evidence base on delayed ASD diagnosis and missed opportunities for early identification in Brazil, identify knowledge gaps, and examine how diagnostic pathways and reported barriers have been described across studies.

## 2. Materials and Methods

### 2.1. Study Design

This scoping review followed the methodological framework of Arksey and O’Malley [14], further refined by Levac et al. [15], and was reported in accordance with PRISMA-ScR [16]. The objective was to map the available evidence on delayed diagnosis and missed opportunities for early autism spectrum disorder (ASD) identification in Brazilian children and adolescents, with emphasis on regional, socioeconomic, and structural determinants, and to identify knowledge gaps in the Brazilian literature. Before protocol development, we searched the published literature for existing reviews on this topic but did not search protocol registries (e.g., PROSPERO or OSF) for similar review protocols. A predefined protocol was prospectively registered on the Open Science Framework (OSF; registration: gtbyj). Eligibility criteria were defined using the Population–Concept–Context (PCC) framework. The population included children and adolescents (0–18 years) residing in Brazil. The concept encompassed ASD screening practices, diagnostic pathways, age at first caregiver concern, age at diagnosis, diagnostic delay, barriers to access, missed opportunities for early identification, and provider training. The context included Brazilian healthcare settings in both the public Unified Health System (SUS) and private services across all regions. Consistent with scoping review methodology, formal methodological quality assessment was not used to exclude studies.

### 2.2. Types of Evidence Sources

Eligible sources included quantitative observational studies (cross-sectional, cohort, retrospective), qualitative and mixed-methods studies, and intervention studies related to diagnostic pathways or provider training. The grey literature (theses and dissertations) was also considered. Studies not conducted in Brazil, prevalence-only studies without diagnostic pathway data, genetic or neurobiological studies, and intervention efficacy trials unrelated to diagnosis were excluded. Review articles were excluded from evidence synthesis but screened to identify additional primary studies and provide contextual background. No date restrictions were applied. Articles in English, Portuguese, or Spanish were included.

### 2.3. Information Sources and Search Strategy

A comprehensive search was conducted in PubMed/MEDLINE, SciELO, LILACS (via BVS) and Scopus. These databases were selected to prioritize international biomedical coverage and the Brazilian/Latin American regional literature. Controlled vocabulary (MeSH/DeCS) and free-text terms related to ASD diagnosis, screening, access to care, and socioeconomic and racial inequalities were used. The equity-related concept block was intentionally retained because regional, socioeconomic, and structural determinants constituted a predefined component of the review question rather than a secondary post hoc analysis. The resulting search therefore prioritized studies capable of informing both diagnostic pathways and the equity-related dimensions specified in the review objectives. The final search was conducted on 30 January 2026. Furthermore, an additional search of the CAPES Theses and Dissertations Database was conducted using terms related to autism spectrum disorder, diagnosis, early identification, screening, and Brazil. No additional eligible studies were identified. Full strategies are provided in Supplementary Annex SI.

**Figure 1:**
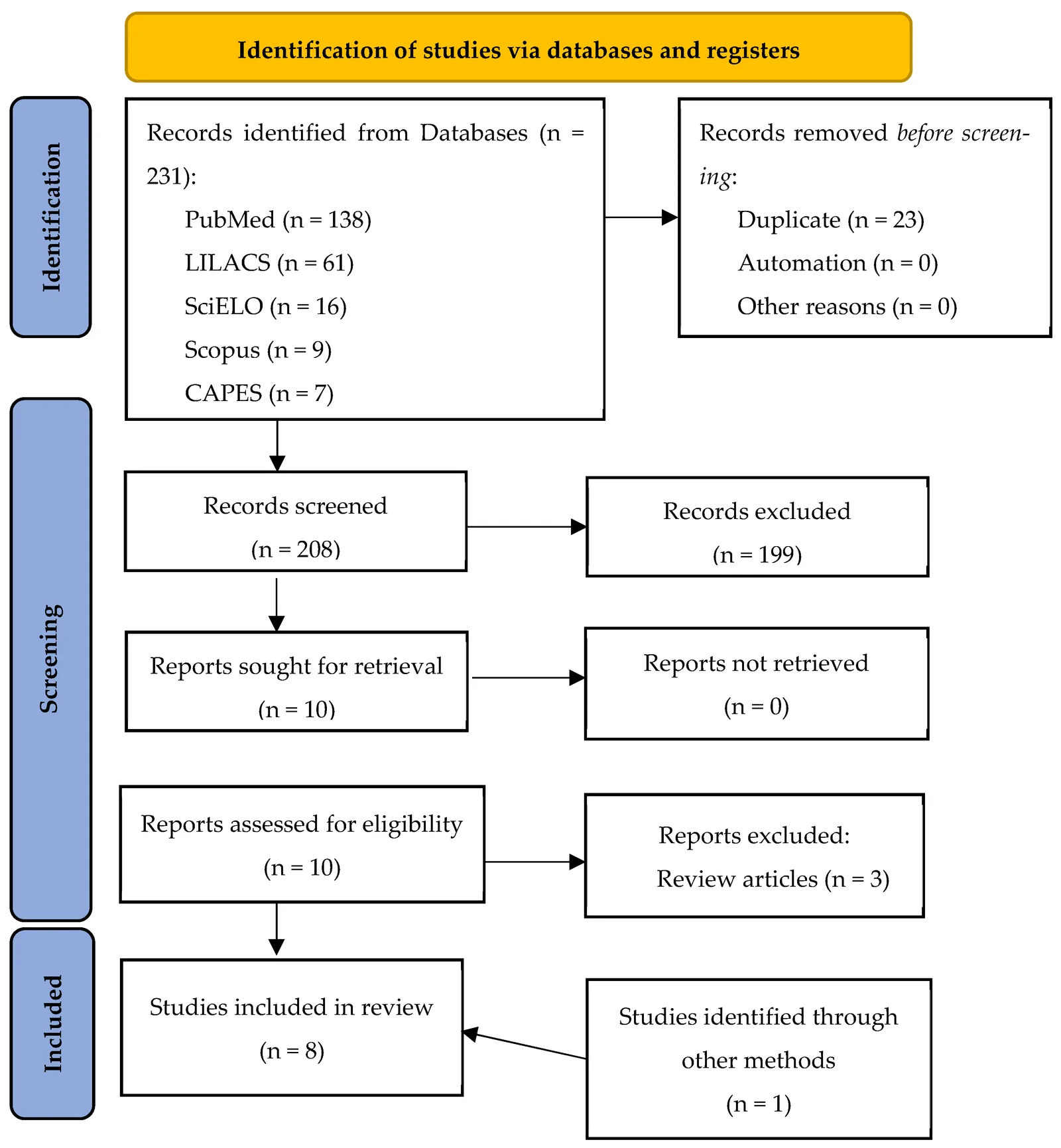
PRISMA-ScR flow diagram of the study selection process.

Records were exported to reference management software Zotero version 90.0.6, and duplicates were removed. Titles and abstracts were screened independently by two reviewers (BTS, MEOH) using Rayyan, followed by full-text assessment. Disagreements were resolved by consensus or third-reviewer adjudication. One additional study was identified through citation searching. The selection process is shown in the PRISMA-ScR flow diagram (Figure 1).

### 2.4. Data Charting (Data Extraction)

Data were extracted by two independent reviewers using a standardized Microsoft Excel data extraction form. Extracted items included study characteristics, sample size, region, and participant characteristics (age, sex, socioeconomic indicators, parental education, and race/ethnicity, when available). Clinical variables included age at first concern, age at diagnosis, definition of diagnostic delay, screening tools, referral pathways, and diagnostic setting. System-level variables included physician training, screening protocols, specialist access, waiting times, and structural inequalities. Barriers were classified using a predefined conceptual framework comprising family-, provider-, service/system-, structural-, and cultural-level domains. Individual barriers were identified from the included studies and assigned to these categories, with additional barriers incorporated when they emerged during data extraction. Missed opportunities were operationally defined as points along the diagnostic pathway where earlier screening, referral, or diagnostic evaluation could reasonably have occurred but did not. Data extraction and barrier classification were performed independently by two reviewers. Before full data extraction, both reviewers independently piloted the extraction form on two studies and refined the form by consensus. Minor discrepancies were resolved through discussion during a consensus meeting, and the final extracted dataset was reviewed by a third reviewer.

### 2.5. Risk of Bias Assessment

Although formal risk-of-bias assessment is not required for scoping reviews, methodological quality was descriptively appraised to support interpretation. Methodological appraisal was conducted independently by two reviewers. Quantitative observational studies were assessed using a predefined adapted Newcastle–Ottawa Scale (NOS), the quasi-experimental study using ROBINS-I, and qualitative studies using the JBI Critical Appraisal Checklist for Qualitative Research. Final assessments were concordant and informed interpretation but were not used for study exclusion.

## 3. Results

The search strategy identified eight studies that met the eligibility criteria for inclusion in this exploratory scoping review. Study characteristics are summarized in Table 1, and key diagnostic timing and pathway data are presented in Table 2. Detailed study objectives and eligibility criteria are provided in Supplementary Table S1, while extended methodological appraisal and contextual data are available in Supplementary Tables S2–S6.

### 3.1. Study Characteristics

Of the eight included studies (Table 1), four were cross-sectional quantitative analyses, three were qualitative studies, and one was a pilot intervention evaluating professional training. Most studies were conducted in the Southeast region, although two large datasets included all five Brazilian regions.

Sample sizes varied substantially, ranging from small qualitative samples (N = 9–21 caregivers) to large administrative datasets (N > 22,000 children from CAPSi services). Across studies, samples were predominantly male (approximately 80–97%), broadly consistent with established ASD sex distribution patterns; however, one study reported a markedly higher male proportion (97.3%), which may reflect referral or selection bias in addition to underlying sex differences in ASD presentation.

### 3.2. Methodological Appraisal

Overall, the included studies demonstrated moderate to high methodological quality according to their respective appraisal tools. Girianelli et al. [5], based on a national administrative dataset, demonstrated the strongest epidemiological reliability and the lowest risk of selection bias among the observational studies. However, all cross-sectional studies were inherently limited in their ability to support causal inference, and recall bias was a concern in caregiver-based surveys. The qualitative studies were generally of moderate to high methodological quality and provided valuable contextual insights into diagnostic pathways, although transferability was limited by single-site or regional recruitment. The pilot intervention study [9], appraised using ROBINS-I, demonstrated improvements in provider knowledge but was limited by the absence of a control group and long-term follow-up, resulting in moderate risk of bias. A summary is provided in Supplementary Table S3 and Supplementary Annex SIII.

### 3.3. Evidence on Early ASD Identification and Diagnostic Pathways

#### Age at Diagnosis and Diagnostic Delay

Mean age at diagnosis varied considerably across studies (Table 2 and Supplementary Table S4). In a national CAPSi dataset [5], the mean age at diagnosis was 66 months (5.5 years), with only 30.4% classified as early diagnoses (<48 months). In a Brazilian subsample of a multinational survey [18], the mean age at diagnosis was 47.3 months (~3.9 years), with a mean delay of 27 months between first caregiver concern and diagnosis. Longer delays were observed in qualitative and tertiary-care studies. One study reported a mean delay of 36 months between first caregiver concern and formal diagnosis [19], while a recent retrospective clinical study [22] identified a mean diagnostic age of 79.2 months (6.6 years), corresponding to an approximate delay of 49 months. Despite methodological variation, the included studies reported delays in ASD diagnosis or diagnostic pathways, although the magnitude and operational definitions varied across studies.

### 3.4. Referral Pathways and Health System Context

Diagnosis was predominantly specialist-driven, most commonly involving neurologists or psychiatrists, with minimal participation of PHC providers in initial diagnosis (Table 2). In one multinational study that included a Brazilian subsample, reliance on the public health system (SUS) was associated with later diagnosis in the overall regression analysis [18]. Referral pathways were often complex and fragmented, typically involving multiple steps such as school-based identification, repeated pediatric consultations, and eventual referral to tertiary care. Qualitative data further highlighted patterns of repeated consultations and geographic barriers, including travel to state capitals to access specialized services. The pilot training intervention [9] demonstrated improved ASD knowledge among PHC providers and increased referral rates, representing the only included study evaluating a primary care training intervention.

### 3.5. Barriers and Missed Opportunities

Barriers were identified across multiple levels and are detailed in Supplementary Table S5. At the family level, these included normalization of early symptoms, financial constraints, transportation difficulties, and caregiver burden. At the provider level, barriers included dismissal of caregiver concerns, inadequate developmental surveillance, limited ASD-specific training, and failure to apply screening tools. At the system level, long waiting times, specialist shortages, absence of routine screening protocols, and fragmented care pathways were often reported. Several included studies described structural inequities, while one national administrative study reported later diagnosis in the North region [5]. Qualitative studies additionally described geographic barriers to accessing specialist services.

The included studies described missed opportunities at multiple points along the diagnostic pathway, including failure to act on early caregiver concerns, absence of screening at initial contact, delayed referral following clinical suspicion, and prolonged intervals before specialist confirmation.

### 3.6. Characteristics of the Available Evidence

#### 3.6.1. Evidence Characteristics: Tools, Settings, Populations, Outcomes, and Operational

##### Definitions

As summarized in Table 1 and Table 2 and Supplementary Table S4, the included studies investigated ASD diagnosis across community, PHC, specialty, tertiary, and mixed healthcare settings. Most studies involved public (SUS) services, although mixed public/private settings were also represented. Study populations included children and adolescents with ASD, caregivers, and PHC providers. Physician involvement ranged from general practitioners to neurologists and psychiatrists, with specialist-led diagnosis predominating. Sociodemographic variables examined across studies included sex, socioeconomic indicators, and parental educational level. Sex was consistently reported, whereas socioeconomic measures varied considerably, including household socioeconomic status, municipal Human Development Index (HDI), caregiver employment, school type, and parental education.

ASD diagnosis was most commonly based on established diagnostic frameworks, including DSM-IV, DSM-5, DSM-5-based clinical diagnosis, and ICD-10 criteria (Supplementary Table S1). Five of the eight included studies explicitly examined screening practices or diagnostic pathways, whereas the remaining studies [5,17,18] focused primarily on diagnostic timing, service utilization, or caregiver experiences without detailing screening pathways. When clinical tools were reported [19,22], they included the Autism Behavior Checklist (ABC), Autism Screening Questionnaire (ASQ), Child Behavior Checklist (CBCL), and the Brazilian Autism Trait Assessment (ATA); however, several studies did not specify the screening or diagnostic instruments used. The principal outcomes investigated included age at diagnosis, diagnostic delay, referral pathways, barriers to diagnosis and treatment access, service utilization, provider training, and factors associated with diagnostic timing. Referral pathways varied considerably and included PHC referral, spontaneous demand, school-based referral, pediatric consultation, specialist assessment, CAPSi referral, and caregiver-initiated help-seeking.

Definitions of diagnostic delay varied across studies. One study classified diagnosis as early (<48 months) or late (≥48 months), whereas others defined delay as the interval between first caregiver concern and formal diagnosis. Several studies reported age at diagnosis without applying an explicit operational definition of diagnostic delay. Across studies, missed opportunities were identified at multiple points along the diagnostic pathway, including dismissal of caregiver concerns, inadequate developmental surveillance, failure to apply screening methods, delayed referral following clinical suspicion, multiple consultations without ASD suspicion, prolonged waiting times for specialist assessment, and delayed diagnostic confirmation. Several studies also identified limited implementation or failure to apply screening methods as provider- or system-level barriers, although few explicitly described the screening instruments used.

### 3.7. Sociodemographic and Regional Findings

#### Regional Disparities

Evidence for regional differences in diagnostic timing was derived primarily from one national administrative study [5], which reported later diagnosis in less-resourced regions, particularly the North. Later diagnoses were observed in less-resourced regions, while specialist services were described as being concentrated in urban centers. The same national study also reported later diagnosis among children receiving care through the public health system (SUS), although this association should be interpreted within the context of that study design [5]. Qualitative studies further described geographic barriers to diagnosis, including families travelling to state capitals to obtain specialist assessment (Supplementary Table S4).

### 3.8. Sex and Gender

Evidence regarding the association between sex and diagnostic timing was limited (Supplementary Table S4). In one included study, neither the sex of the autistic individual nor caregiver educational level significantly predicted age at diagnosis [18]. However, study participants were predominantly male (>80%) across the included studies, limiting assessment of sex-related differences. Girianelli et al. [5] also discussed greater perceived barriers to accessing diagnostic services among caregivers of female preschool children (Supplementary Table S5), suggesting that sex- and gender-related factors may influence caregiver perceptions of access to diagnostic services, although evidence regarding sex differences in diagnostic timing remains limited.

### 3.9. Socioeconomic Status, Parental Education, and Race/Ethnicity

Evidence regarding socioeconomic factors was limited and inconsistent across the included studies (Supplementary Table S4). Study populations ranged from predominantly middle- to high-socioeconomic-status families to cohorts largely receiving care through the public health system (SUS) or enrolled in public schools. One national study found no significant association between municipal Human Development Index (HDI) and early diagnosis after multivariable analysis [5]. Information on parental educational level was reported descriptively in some studies but was not consistently examined in relation to diagnostic timing. Race and ethnicity were rarely reported. Only one included study extracted race/skin color data, but approximately 39% of records had missing information [5], precluding meaningful analysis of racial and ethnic differences in diagnostic timing.

An appraisal of the certainty and limitations of the evidence is presented in Supplementary Table S2 and Supplementary Annex SII.

## 4. Discussion

### 4.1. Magnitude of Diagnostic Delay: Brazilian Findings and International Context

Overall, the available evidence suggests that delayed ASD diagnosis in Brazil is influenced by multiple interacting factors, including prolonged diagnostic intervals, limited implementation of early screening, concentration of specialists in urban centers, and reported regional and socioeconomic inequities. Collectively, the available evidence points to health system factors as important contributors to diagnostic delay, although the small and heterogeneous evidence base limits definitive conclusions.

When integrated with prior Brazilian and international reviews [2,12,13], the present review suggests that delayed ASD diagnosis in Brazil extends beyond issues of awareness and reflects multilevel health system factors. A comparison between the present review and previous reviews is provided in Supplementary Table S6. Framing diagnostic delay through a “missed opportunities” perspective may provide a useful way of organizing the available evidence and identifying priorities for future research and health system improvement (Supplementary Table S5).

Across the included Brazilian studies, age at ASD diagnosis and diagnostic intervals varied substantially, although all reported delays in diagnosis or prolonged diagnostic intervals [5,18,19,22]. Population-based data suggested relatively earlier diagnoses, whereas tertiary-care and qualitative samples reflected more pronounced delays, often accompanied by prolonged intervals between first caregiver concern and formal diagnosis.

Consistent with international findings [2], the included Brazilian studies reported substantial diagnostic delay. Tertiary-care samples generally showed longer delays than population-based studies, suggesting that referral complexity and access to specialized services may contribute to prolonged diagnostic pathways [22].

For comparison, a global meta-analysis reported a pooled mean age at ASD diagnosis of 60.48 months, with estimates across countries ranging from 30.90 to 74.70 months [4]. While Brazilian estimates generally fall within this international range, findings from tertiary-care samples tend to exceed pooled global averages, which may reflect more complex referral pathways and delayed access to specialized services.

Importantly, previous studies emphasize that reductions in mean age at diagnosis do not necessarily reflect improvements in the interval between first caregiver concern and confirmed diagnosis, which often remains prolonged. Although definitions of diagnostic delay vary across the literature [3], this lack of definitional clarity complicates cross-country comparisons and underscores the importance of examining diagnostic trajectories, particularly the interval between first concern and formal diagnosis, rather than relying solely on mean age benchmarks. Consistent with the broader findings of Matos et al. [2], the present review further highlights intra-country disparities within Brazil. These observations indicate that, beyond national income classification, regional and structural inequalities may play a particularly important role in shaping diagnostic trajectories within heterogeneous contexts.

These findings suggest differences across levels of care and that delays may accumulate along the diagnostic pathway, particularly in cases requiring referral to specialized services. The observed variability also likely reflects heterogeneity in access to care, service organization, and referral processes across settings.

The persistence of extended intervals between first concern and diagnosis suggests that delayed symptom recognition alone may not fully explain the prolonged diagnostic intervals reported across studies and that health system factors may also contribute. Collectively, these findings support considering diagnostic delay as a process involving multiple stages of the diagnostic pathway.

Earlier reviews identified multilevel predictors of earlier ASD identification, many of which were embedded within health system organization rather than clinical presentation alone [7]. The present review extends this work by contextualizing these mechanisms within the Brazilian health system and by mapping missed opportunities across the diagnostic pathway.

Similarly, an international systematic review reported that greater symptom severity, stronger parental concern, and higher socioeconomic status were associated with earlier ASD identification, whereas referral pathways and access to specialized services were less consistently examined [8]. Similar determinants were described across the Brazilian studies included in this review, particularly regarding symptom recognition, referral coordination, and service organization.

Together, these findings suggest that many determinants of diagnostic timing described internationally are also reported in Brazil, while highlighting the importance of understanding how they operate within the Brazilian health system.

### 4.2. Structural and Regional Inequities Within Brazil

Previous Brazilian reviews have highlighted the concentration of wealth and healthcare resources in the South and Southeast regions [12] but have not consistently quantified its impact on diagnostic timing. Regional disparities in diagnostic timing were primarily identified in one national administrative study, while qualitative studies described geographic barriers consistent with unequal access to specialist services. Similar patterns have been described in U.S. healthcare systems, where the “diagnostic odyssey” is prolonged by reliance on subspecialty teams and extended waiting times [23]. Notably, in the United States, the mean age at diagnosis continues to exceed 48 months despite the availability of validated screening tools and early-intervention mandates, suggesting that delays may be influenced not only by resource availability but also by how services are organized [23].

These findings suggest an important role of tertiary psychosocial centers in absorbing neurodevelopmental diagnoses that may not have been identified or managed earlier within PHC, particularly in less resourced regions where specialist access is centralized. The association between public health system utilization (SUS) and later diagnosis further highlights inequities within Brazil’s health system.

This pattern aligns with broader epidemiological evidence showing that income inequality in Brazil remains strongly associated with morbidity patterns across non-communicable diseases, even after adjustment for healthcare access and socioeconomic covariates [11]. Within this context, delayed ASD diagnosis may be better understood as embedded within wider structural gradients rather than as an isolated clinical inefficiency.

Thus, while the earlier literature emphasized socioeconomic hardship and caregiver burden [13], the included studies place these experiences within a broader context of workforce shortages, long waiting times, and referral bureaucracy.

Previous analyses of CAPSi utilization have also reported marked regional variation in specialized autism-related services and limitations in routinely collected race/skin color data [10,24], supporting the regional disparities identified in the present review.

### 4.3. Screening, Referral Pathways, and Implementation Gaps

Sukiennik et al. [12] highlighted limitations in the translation, validation, and implementation of diagnostic and screening tools in Brazil. The included studies suggest that an important bottleneck may lie not in the availability of screening instruments, but in their routine integration into PHC practice. Although national guidelines recommend early identification, developmental surveillance was inconsistently reported across the included studies.

Across the included studies, reported barriers suggest that workforce training, referral coordination, and health system organization remain important implementation challenges.

Comparable implementation challenges have also been observed internationally; in U.S. community settings, awareness and screening initiatives increased referral rates but did not consistently reduce age at confirmed diagnosis in the absence of coordinated follow-up systems [25].

Qualitative evidence described by Gomes et al. [13] highlighted parental distress associated with diagnostic delays, repeated consultations with multiple health professionals, and difficulties in accessing services. The present review similarly identified relational and communication barriers within Brazilian diagnostic pathways.

By mapping these events across the diagnostic trajectory, the proposed missed opportunity framework organizes provider- and system-level events described across the included studies.

### 4.4. Primary Health Care as a Leverage Point

Several included studies highlighted PHC as an important component of the diagnostic pathway. Bordini et al. [9] demonstrated that brief training significantly increased provider knowledge and referrals. Together, these findings suggest that PHC may represent an important leverage point for improving earlier identification. Despite the central role of PHC, ASD diagnosis remained predominantly specialist-driven, suggesting that opportunities for earlier identification within primary care may not yet be fully realized [18,19]. Strengthening developmental surveillance within PHC may represent one potential strategy to reduce diagnostic delay. The included studies suggest that specialist scarcity alone may not fully explain diagnostic delays and that structured support at the primary care level may also be important.

Comparable implementation approaches have also been described internationally. A recent U.S.-based scoping review of ASD diagnosis in PHC examined the feasibility of primary care provider (PCP)-led diagnosis, demonstrating improved diagnostic agreement and reduced wait times in selected contexts [26]. This review focused primarily on intervention implementation and provider-level outcomes rather than sociodemographic disparities. The PHC intervention literature itself, however, remains limited to small-scale feasibility models. In contrast, the present review places these findings within the broader context of structural, regional, and socioeconomic factors described in the Brazilian literature.

### 4.5. Sex and Gender Considerations in Diagnostic Timing

The limited evidence available regarding sex differences in diagnostic timing should be interpreted cautiously, particularly given the predominance of male participants across the included studies and the potential underrepresentation of female presentations. Although the Brazilian evidence was limited, these findings are consistent with the broader national and international literature suggesting possible under-recognition of ASD in girls [27,28].

Recent Brazilian psychometric research has directly addressed concerns regarding sex-related diagnostic blind spots. Noting that traditional ASD criteria were largely derived from male-dominated samples, a recent study [27] developed screening instruments designed to capture camouflaging behaviors and gender-related autistic traits. These findings are consistent with concerns that traditional criteria may insufficiently capture female presentations, potentially contributing to delayed or missed identification.

The international literature has similarly described sex-specific differences in symptom presentation, including greater social camouflaging, subtler restricted interests, and higher rates of internalizing symptoms in females, which may contribute to delayed or missed recognition [28]. Qualitative accounts of late-diagnosed adult women further suggest that gendered expectations and compensatory strategies may obscure early developmental concerns from both families and clinicians. Together, these findings are consistent with the broader literature suggesting that subtle or masked presentations may contribute to delayed or missed identification across the lifespan. This area remains underexplored in Brazilian child and adolescent research and represents an important priority for future investigation.

### 4.6. Reframing Diagnostic Delay: A Missed Opportunity Framework

One contribution of this review is the proposal of a structured missed opportunity framework. Earlier reviews have described diagnostic delay primarily in descriptive terms but have not systematically categorized where earlier identification could have occurred. By distinguishing barriers across family, provider, service, structural, and governance levels, the proposed framework provides a way of organizing the available evidence beyond descriptions of delayed diagnosis alone.

This reframing is particularly relevant in the Brazilian context, where national child health policies emphasize routine developmental surveillance and early identification, rather than universal autism-specific screening [12]. Within the available literature, the principal gap appears to be operational rather than normative, reflecting challenges in implementation rather than policy design.

### 4.7. Limitations

Several limitations should be considered when interpreting the findings of this scoping review. Importantly, many of these reflect not only methodological weaknesses within individual studies but also broader structural gaps in the Brazilian research landscape on ASD diagnosis.

First, despite a comprehensive search strategy, only eight heterogeneous studies met the eligibility criteria for inclusion, highlighting the limited and geographically concentrated evidence base available for this topic in Brazil. Several conclusions, particularly regarding regional differences in diagnostic timing, were informed primarily by a single national administrative dataset and should therefore be interpreted cautiously. As a result, the findings may underrepresent diagnostic pathways in rural, remote, and under-resourced regions, where specialist scarcity and referral fragmentation may be more pronounced, thereby constraining national-level generalizability.

Substantial methodological heterogeneity characterized the included studies, limiting comparability and precluding quantitative synthesis. While such heterogeneity justifies the use of a scoping review rather than a meta-analysis, it also highlights the absence of standardized national metrics for monitoring diagnostic timing.

The predominance of cross-sectional and retrospective observational designs further limits causal inference. Caregiver-reported data are subject to recall bias, tertiary-care samples may overestimate delays due to referral filtering, and administrative datasets may underestimate clinical complexity due to limited granularity. Together, these factors restrict the ability to disentangle health system-related delays from those associated with symptom complexity.

Data on race, ethnicity, gender, and intersectional inequities were sparse or inconsistently reported. These gaps reflect broader patterns of underinvestment in equity-focused neurodevelopmental research.

The scarcity of implementation studies examining the consistent application of screening protocols, referral coordination, and health system integration limits the ability to evaluate scalable solutions. Consequently, although provider- and system-level barriers were consistently described, the strength of evidence supporting specific intervention pathways remains limited.

Limitations inherent to the scoping review design should also be acknowledged. This review aimed to map the breadth and characteristics of available evidence rather than to estimate pooled effect sizes. In addition, unpublished program evaluations and regional health department data may not have been captured. Although the grey literature was eligible and an additional search of the CAPES Theses and Dissertations Database was conducted, no eligible studies were identified through this search, and other unpublished Brazilian sources may still have been missed. Finally, because the electronic search combined terms for diagnostic pathways with equity-related terms using AND, studies addressing diagnostic delay, screening, or referral pathways that did not explicitly report equity-related concepts in searchable fields may not have been retrieved.

Taken together, these limitations indicate that the findings of this review should be interpreted as mapping patterns reported across the available literature rather than supporting definitive conclusions about diagnostic pathways at the national level. At the same time, the fragmentation, geographic concentration, and methodological variability of the evidence base highlight important gaps in research on early ASD identification in Brazil. These findings also point to the need for more standardized, longitudinal, and equity-sensitive research to better understand diagnostic pathways and inform future health system improvements.

### 4.8. Future Research Directions

The findings of this exploratory scoping review highlight several priorities for future research on ASD identification in Brazil. First, there is a need for larger, methodologically standardized studies with broader geographic representation, particularly in the North, Northeast, and other underrepresented regions. Nationally comparable measures of age at diagnosis, diagnostic delay, and referral pathways would facilitate more robust monitoring of diagnostic practices over time.

Second, implementation research is needed to evaluate strategies for integrating developmental surveillance and ASD screening into routine PHC. Although national guidelines recommend early identification, few studies have examined the real-world implementation, acceptability, and effectiveness of screening and referral pathways within the Brazilian health system.

Third, prospective longitudinal studies following children from first caregiver concern through diagnostic confirmation would improve understanding of where delays accumulate along the diagnostic pathway and help distinguish family-, provider-, and system-level contributors to delayed diagnosis.

Finally, future research should prioritize equity-sensitive approaches by consistently reporting race and ethnicity, socioeconomic indicators, parental education, and geographic characteristics. Greater attention to underserved populations, female presentations of ASD, and regional differences in service organization will be essential to better understand and reduce disparities in timely identification.

## 5. Conclusions

This exploratory scoping review mapped the available evidence on delayed ASD diagnosis and missed opportunities for early autism identification in Brazil. Although the included studies varied substantially in design, setting, and methodological quality, they consistently described prolonged diagnostic pathways and multiple reported barriers to timely diagnosis. Building on the available evidence, the proposed missed opportunity framework provides a way of organizing reported barriers across different stages of the diagnostic pathway and may help guide future research and implementation efforts.

Collectively, the included studies suggest that strengthening PHC, developmental surveillance, and referral coordination may represent promising areas for future investigation and service improvement.

Overall, this review highlights important knowledge gaps regarding diagnostic pathways and early ASD identification in Brazil. Future research, particularly studies using standardized methods and broader geographic representation, will be essential to better understand diagnostic pathways and diagnostic delay and inform evidence-based policy and practice.

## Figures and Tables

**Table 1:** Characteristics of included studies.

Study	Authors (Year)	Design	Population	Sample Size (N)
1	Araripe et al. (2022) [17]	Cross-sectional	Caregivers	1200 (analytic: 927)
2	Girianelli et al. (2023) [5]	Cross-sectional	Children	23,657 (analytic: 22,483)
3	Montiel-Nava et al. (2024) [18]	Cross-sectional	Caregivers	2520 (Brazil subsample: 1000)
4	Ribeiro et al. (2017) [19]	Qualitative	Caregivers	19 mothers (19 children)
5	Riccioppo et al. (2024) [20]	Qualitative	Caregivers	21 (analytic: 20)
6	Bonfim et al. (2020) [21]	Qualitative	Caregivers	9 relatives (8 children)
7	Bordini et al. (2015) [9]	Pilot intervention	PHC providers	29 (analytic: 22)
8	Augusto et al. (2025) [22]	Retrospective clinical	Children/adolescents	106 eligible (ASD: 37)

**Table 2:** Diagnostic timing and pathways across included studies.

Study	Age at Diagnosis	Delay (Months)	Setting	Region	Diagnostic Care Pathway
1	NR	NR	Community	National	Specialist-based care
2	66.0 (SD 34.8)	NR	Specialty (public)	National	PHC and multiple entry points; PHC associated with earlier diagnosis
3	47.3 (SD 30.5)	27.0	Specialty	Brazil (multinational sample)	Mixed (generalist and specialist)
4	59.6 (SD 40.5)	36.0	Tertiary	Southeast	Pediatrician → delayed referral → specialist diagnosis
5	NR	NR	Community/specialty	Southeast	Caregiver-led pathway with delayed help-seeking
6	NR	NR	PHC/specialty	Midwest	School concern → referral → multiple consultations
7	NR	NR	PHC/specialty	Southeast	PHC suspicion → referral to CAPSi
8	79.2 (SD 32.4)	49.3	Tertiary	Southeast	Specialist-driven (neurology predominant)

NR: Not Reported.

## Data Availability

The data presented in this study are included in the article and Supplementary Materials.

## Supplementary Materials

The following supporting information can be downloaded at https://www.mdpi.com/article/10.3390/children13081082/s1, Supplementary Annex SI: Complete Electronic Search Strategy; Supplementary Annex SII: Exploratory appraisal of evidence strength; Supplementary Annex SIII: Narrative Summary of Methodological Appraisal; Supplementary Table S1: Detailed study objectives, inclusion criteria, and diagnostic frameworks of included studies; Supplementary Table S2: Exploratory evidence strength profile; Supplementary Table S3: Methodological appraisal of included studies; Supplementary Table S4: Quantitative and contextual diagnostic data across included studies; Supplementary Table S5: Mapped barriers and missed opportunities in the ASD diagnostic pathway; Supplementary Table S6: Comparative characteristics of reviews related to autism. References [29,30,31] are cited in the supplementary materials.
