# Supplementary material for: Delayed Diagnosis and Missed Opportunities for Early Autism Identification in Brazil: An Exploratory Scoping Review
Source: Children (Basel). 2026 Aug 15;13(8):1082. doi: 10.3390/children13081082 (PMC13510629; doi:10.3390/children13081082)
Supplement: Supplementary file 1 [file children-13-01082-s001.zip › children-4457266-supplementary.pdf]

Supplementary Materials

Supplementary Annex SI. Complete Electronic Search Strategy

PubMed

("Autism Spectrum Disorder"[MeSH] OR "Autistic Disorder"[MeSH] OR "autism" OR "ASD" OR "TEA") AND (Brazil* OR "Brasil*") AND (Screening OR "early identification" OR "diagnosis" OR "delayed" OR "late diagnosis" OR "barriers" OR "pathway" OR "access") AND ("socioeconomic" OR "income" OR "poverty" OR "vulnerability" OR "education" OR "schooling" OR "race" OR "ethnicity" OR "Afro-descendant" OR "Pardo" OR "Black" OR "inequality" OR "disparity")

Scopus

("Autism Spectrum Disorder"[MeSH] OR "Autistic Disorder"[MeSH] OR "autism" OR "ASD" OR "TEA") AND (Brazil* OR "Brasil*") AND (Screening OR "early identification" OR "diagnosis" OR "delayed" OR "late diagnosis" OR "barriers" OR "pathway" OR "access") AND ("socioeconomic" OR "income" OR "poverty" OR "vulnerability" OR "education" OR "schooling" OR "race" OR "ethnicity" OR "Afro-descendant" OR "Pardo" OR "Black" OR "inequality" OR "disparity")

LILACS

(tw:(autismo OR "transtorno do espectro autista" OR TEA OR autism)) AND (tw:(Brasil OR Brazil)) AND (tw:(diagnóstico OR "identificação precoce" OR rastreamento OR triagem OR barreiras OR acesso OR screening OR diagnosis)) AND (tw:(socioeconômico OR escolaridade OR raça OR cor OR etnia OR desigualdade OR vulnerabilidade OR "afro-brasileiro" OR pardo OR preto OR "norte" OR "sul"))

SciELO

(tw:(autismo OR "transtorno do espectro autista" OR "TEA" OR "autism")) AND (tw:(Brasil OR Brazil OR brasileiro OR Brazilian)) AND (tw:(diagnóstico OR "diagnóstico tardio" OR "identificação precoce" OR "rastreamento" OR "triagem" OR "barreiras" OR "acesso" OR "diagnosis" OR "delayed diagnosis" OR "screening" OR "barriers")) AND (tw:(socioeconômico OR renda OR escolaridade OR raça OR cor OR etnia OR desigualdade OR vulnerabilidade OR "afro-brasileiro" OR "pardo" OR "preto" OR "norte" OR "sul" OR "socioeconomic" OR "income" OR "schooling" OR "race" OR "ethnicity" OR "inequality" OR "vulnerability"))

CAPES

"transtorno do espectro autista" OR autismo OR TEA AND Brasil OR brasileiro OR brasileira AND diagnóstico OR "diagnóstico precoce" OR "diagnóstico tardio" OR "identificação precoce" OR rastreamento OR triage AND barreiras OR acesso OR encaminhamento OR "atenção primária" OR "atenção básica"

Supplementary Annex SII - Exploratory appraisal of evidence strength

The exploratory appraisal provided a contextual interpretation of the strength and limitations of the mapped evidence. The resulting evidence strength profile (Supplementary Table S2) suggests overall low to moderate confidence across key outcomes. The appraisal considered methodological quality, consistency, directness, precision, and publication bias when judging the overall strength of the available evidence. These domains were used as an exploratory framework to structure interpretation of the mapped evidence and were not intended to constitute a formal GRADE certainty assessment. Domain judgments were based on the characteristics of the contributing evidence: methodological quality reflected the design-specific appraisal findings; inconsistency reflected the degree of agreement across contributing studies; indirectness reflected the applicability of the evidence to the review question and Brazilian context; imprecision reflected limitations related to sample size, number of contributing studies, and uncertainty of estimates; and publication bias reflected whether the available evidence suggested potential selective availability of findings. Overall evidence strength was determined by considering these domains together, with greater limitations across domains resulting in lower overall ratings. Ratings of “Very Low,” “Low,” “Low–Moderate,” and “Moderate” were used descriptively to distinguish the relative strength of the mapped evidence and should not be interpreted as formal GRADE certainty categories. Because most included quantitative studies were observational (cross-sectional or retrospective) and qualitative studies contributed contextual evidence, overall confidence in the available evidence remained limited.

Key outcomes evaluated included age at ASD diagnosis in Brazil; diagnostic delay (first concern to diagnosis interval); regional disparities (North vs. South/Southeast); socioeconomic disparities (SUS vs. private coverage and SES proxies); provider-level barriers (training, developmental surveillance, and screening use); impact of provider training interventions; and gender-related diagnostic disparities.

Overall, the strength of the available evidence ranged from low to moderate across the evaluated outcomes. The available evidence consistently supported delayed ASD diagnosis, while evidence regarding regional disparities was derived primarily from one national administrative study and was complemented by qualitative evidence on geographic barriers to specialist access. Evidence regarding socioeconomic and gender inequities, as well as intervention effectiveness, was comparatively weaker because of methodological limitations and heterogeneity across studies. The exploratory appraisal identified strengths, including the use of a large national administrative dataset, consistent identification of delayed diagnosis, and convergent qualitative mechanisms. Limitations include reliance on observational designs, absence of prospective longitudinal data, limited adjustment for SES and ethnicity, overrepresentation of the Southeast region, and scarcity of screening implementation studies.

Supplementary Annex SIII - Narrative Summary of Methodological Appraisal

Methodological quality was appraised using design-specific critical appraisal tools and summarized narratively. Methodological appraisal of quantitative observational studies was conducted using an adapted Newcastle–Ottawa Scale (NOS) [29]. The adapted tool evaluated three domains: Selection (maximum 5 stars), Comparability (maximum 2 stars), and Outcome (maximum 3 stars), for a total possible score of 10 stars. Overall methodological quality was classified as high (8–10 stars), moderate (5–7 stars), or low (0–4 stars). Qualitative studies were appraised using the JBI Critical Appraisal Checklist for Qualitative Research [30], which evaluates 10 criteria covering methodological congruity, researcher positioning and reflexivity, representation of participants, ethical conduct, and consistency between analysis and conclusions. Items were rated as Yes, No, Unclear, or Not applicable. Overall methodological quality was classified according to the number of criteria rated "Yes" as high (8–10), moderate (5–7), or low (0–4). The quasi-experimental study was appraised using ROBINS-I [31] across seven domains: confounding, selection of participants, classification of interventions, deviations from intended interventions, missing data, measurement of outcomes, and selection of the reported result. Domain-level judgments were classified as low, moderate, serious, or critical risk of bias, or no information, with the overall judgment derived according to ROBINS-I guidance. Appraisal was conducted independently by two reviewers, with disagreements resolved by consensus.

Overall, most studies were judged to have moderate to high methodological quality according to their respective appraisal tools. The qualitative studies were generally judged to be of high methodological quality and provided valuable contextual insights into barriers to early autism identification. The national administrative data [1] provide the strongest epidemiological reliability among the included studies.

The adapted NOS was applied to observational studies (codes 1, 2, 3, and 8). Girianelli et al. (2023), based on a national administrative dataset, demonstrated lower risk of selection bias. However, all cross-sectional studies are inherently limited in causal inference. Recall bias was a concern in caregiver-based surveys [2,3]. These analyses are summarized in Supplementary Table S3.

The three qualitative studies (codes 4, 5, and 6) were appraised using the JBI Critical Appraisal Checklist for Qualitative Research. Ribeiro et al. (2017) met 7 of 10 criteria and was classified as moderate methodological quality, while Riccioppo et al. (2024) and Bonfim et al. (2020) met 8 and 9 criteria, respectively, and were classified as high methodological quality. The principal weaknesses concerned researcher positioning and reflexivity, while participant representation, ethical conduct, and congruity between the qualitative methods, analysis, and interpretation were generally adequate (Supplementary Table S3).

The pilot intervention study (code 7) [4] was appraised using ROBINS-I and was judged to have moderate overall risk of bias. Moderate risk-of-bias judgments were assigned for confounding, selection of participants, measurement of outcomes, and selection of the reported result, while classification of interventions, deviations from intended interventions, and missing data were judged to have low risk of bias. The absence of any domain judged

as serious or critical resulted in an overall moderate risk-of-bias judgment (Supplementary Table S3).

Common methodological limitations across studies included tertiary-care overrepresentation (Southeast concentration), predominantly male samples (reflecting epidemiology but potentially under-detecting females), limited adjustment for socioeconomic confounders, and absence of prospective longitudinal designs.

Supplementary Table S1. Detailed study objectives, inclusion criteria, and diagnostic frameworks of included studies

Code	Authors	Objective(s)	Inclusion criteria	ASD definition / Diagnostic framework	Screening /pathway described? (Y/N)
1	Araripe et al. (2022)	1. Describe the profile of health and education service use; 2. Describe barriers to access; 3. Identify sociodemographic factors associated with treatment access and barriers	Caregivers of children and adolescents with ASD (3–17 years)	DSM-5	No
2	Girianelli et al. (2023)	To investigate factors associated with early diagnosis of autism and other pervasive developmental disorders (PDD) in children treated at CAPSi in Brazil (2013–2019).	Children aged 1–12 years; first CAPSi appointment between 2013–2019; ICD-10 diagnoses: F84, F84.0, F84.1, F84.8, F84.9	ICD-10	No
3	Montiel-Nava et al. (2024)	Identify factors associated with age of ASD diagnosis (and the gap from first caregiver concern to diagnosis) among caregivers of autistic children in Latin America/Caribbean countries; Brazil subsample extracted here.	For Brazil subsample Caregiver or parent of an autistic individual; Resident in Brazil; Caregiver age ≥18; Child age 2–17; Reported a DSM-based autism-related diagnosis in the survey	DSM-IV, DSM-5	No
4	Ribeiro et al. (2017)	To evaluate the pathway between initial parental concerns about atypical child development and ASD diagnosis in Brazil.	Child with confirmed ASD diagnosis; recruited from specialty ASD clinic at UNIFESP (São Paulo)	DSM-IV	Yes
5	Riccioppo et al. (2024)	To explore lived experiences related to autism among families/participants rather than diagnostic timing.	Primary family caregivers (≥18 years) of children aged 0–6 years with an ASD diagnosis (≥6 months), attending: an NGO (“Laço Azul”) and a Child Development Teaching & Research Center (NEPDI), linked to a public higher education institution (UFTM), in Minas Gerais, Brazil; exclusion if comorbidities present.	Not reported	Yes

Code	Authors	Objective(s)	Inclusion criteria	ASD definition / Diagnostic framework	Screening /pathway described? (Y/N)
6	Bonfim et al. (2020)	To describe the family's experience in discovering the diagnosis and initiating treatment of children with ASD	Relative of a child with an ASD diagnosis or diagnostic hypothesis for ≥1 year; age ≥18; involved in direct care; cognitively able to participate.	Not reported	Yes
7	Bordini et al. (2015)	To evaluate the impact of a training program on ASD identification offered to Brazilian health professionals (PHC providers).	Participants were pediatricians and other health professionals selected by local managers who attended the program and completed questionnaires.	DSM-IV	Yes
8	Augusto et al. (2025)	To examine: 1. The time interval between first parental concerns and diagnosis of ASD or ID; 2. Associations between age at diagnosis and cognitive, adaptive, and behavioral functioning.	Diagnosis of ASD; complete assessment in database; exclusion of ADHD and specific learning disorder.	DSM-5, clinical diagnosis (unspecified)	Yes

Supplementary Table S2. Exploratory evidence strength profile

Outcome	Study Designs Contributing	Risk of Bias	Inconsistency	Indirectness	Imprecision	Publication Bias	Overall strength of evidence	Comments
Age at diagnosis	Large cross-sectional + retrospective	Moderate	Low–Moderate	Low	Low	Unclear	**Moderate**	Consistent mean diagnosis >4–5 years; large national dataset strengthens confidence
Diagnostic delay interval	Cross-sectional + qualitative	Moderate	Moderate	Low	Moderate	Unclear	**Low–Moderate**	Directly reported diagnostic delays ranged from approximately 24–36 months; an additional estimate of 49.3 months was calculated from reported means.
Regional disparities	National administrative + survey	Low–Moderate	Low	Low	Low	Unlikely	**Moderate**	Later diagnosis in the North was reported in one national administrative study; qualitative studies additionally described geographic barriers to specialist access
SES disparities	Administrative + survey + clinical	Moderate	Moderate	Moderate	Moderate	Unclear	**Low**	SES often inferred (school type, HDI); not consistently adjusted
Provider-level barriers	Qualitative + survey + intervention	Moderate–Serious	Low	Low	Moderate	Unclear	**Low–Moderate**	Strong thematic consistency but limited high-quality quantitative confirmation
Effect of training intervention	Single pre–post pilot	Moderate	NA	Low	Moderate	Unclear	**Low**	Promising but small sample, no control group
Gender-related disparity	Single quantitative study	Moderate	Serious (single source)	Low	Moderate	Unclear	**Very Low–Low**	Evidence limited to one study; not consistently replicated

Note: This profile represents an exploratory, descriptive appraisal rather than a formal GRADE certainty-of-evidence assessment. Domain and overall ratings were assigned using the decision framework described in Supplementary Annex II.

Supplementary Table S3. Methodological appraisal of included studies

Code	Authors (Year)	Study Design	Appraisal Tool	Overall Methodological Appraisal	Key Concerns
1	Araripe et al. (2022)	Cross-sectional (caregiver survey) **– Quantitative observational**	Adapted NOS	**Moderate methodological quality**	Self-reported service use; SES skew toward middle/high; cross-sectional design
2	Girianelli et al. (2023)	Cross-sectional (national CAPSi dataset) **– Quantitative observational**	Adapted NOS	**High methodological quality**	Administrative data; binary early/late classification; limited variance explained
3	Montiel-Nava et al. (2024)	Cross-sectional (survey; Brazil subsample) **– Quantitative observational**	Adapted NOS	**Moderate methodological quality**	Caregiver recall bias; Brazil subsample within larger regional dataset
4	Ribeiro et al. (2017)	Qualitative (tertiary clinic)	JBI	**Moderate methodological quality**	Single tertiary-care setting
5	Riccioppo et al. (2024)	Qualitative (NGO/teaching center)	JBI	**High methodological quality**	Single regional setting; exclusion of children with comorbidities limits transferability
6	Bonfim et al. (2020)	Qualitative	JBI	**High methodological quality**	Single geographic region
7	Bordini et al. (2015)	Pilot intervention (pre-post)	ROBINS-I	**Moderate risk of bias**	No control group; small sample; short follow-up
8	Augusto et al. (2025)	Retrospective clinical study**– Quantitative observational**	Adapted NOS	**Moderate methodological quality**	Tertiary sample; exclusion criteria may affect representativeness

Supplementary Table S3a. Detailed adapted Newcastle–Ottawa Scale appraisal of quantitative observational studies

Code	Authors (Year)	Selection (0–5★)	Comparability (0–2★)	Outcome (0–3★)	Total Score (0–10★)	Overall Methodological Quality
1	Araripe et al. (2022)	3★	2★	2★	7★	Moderate
2	Girianelli et al. (2023)	4★	2★	2★	8★	High
3	Montiel-Nava et al. (2024)	3★	2★	1★	6★	Moderate
8	Augusto et al. (2025)	3★	1★	2★	6★	Moderate

Note: The adapted Newcastle–Ottawa Scale (NOS) [29] assessed Selection (maximum 5 stars), Comparability (maximum 2 stars), and Outcome (maximum 3 stars), for a maximum total score of 10 stars. For consistency across appraisal tools, overall methodological quality was harmonized into three categories for the present review: high (8–10 stars), moderate (5–7 stars), and low (0–4 stars).

Supplementary Table S3b. Detailed JBI Critical Appraisal Checklist for Qualitative Research

Code	Authors (Year)	Q1	Q2	Q3	Q4	Q5	Q6	Q7	Q8	Q9	Q10	Overall Methodological Quality
4	Ribeiro et al. (2017)	Unclear	Yes	Yes	Yes	Yes	No	No	Yes	Yes	Yes	Moderate
5	Riccioppo et al. (2024)	Yes	Yes	Yes	Yes	Yes	No	No	Yes	Yes	Yes	High
6	Bonfim et al. (2020)	Yes	Yes	Yes	Yes	Yes	Yes	No	Yes	Yes	Yes	High

Note: The JBI Critical Appraisal Checklist for Qualitative Research [30] assessed 10 criteria rated as Yes, No, Unclear, or Not applicable. JBI items: Q1, congruity perspective/methodology; Q2, methodology/research question; Q3, methodology/data collection; Q4, methodology/data analysis; Q5, methodology/interpretation; Q6, researcher culturally/theoretically located; Q7, researcher influence addressed; Q8, participant voices adequately represented; Q9, ethical conduct/approval; Q10, conclusions supported by analysis/interpretation. For consistency across appraisal tools, overall methodological quality was harmonized into three categories for the present review based on the number of criteria rated "Yes": high (8–10), moderate (5–7), and low (0–4).

Supplementary Table S3c. Detailed ROBINS-I appraisal of the quasi-experimental study

Code	Authors (Year)	D1: Confounding	D2: Selection of Participants	D3: Classification of Interventions	D4: Deviations from Intended Interventions	D5: Missing Data	D6: Measurement of Outcomes	D7: Selection of Reported Result	Overall Risk of Bias
7	Bordini et al. (2015)	Moderate	Moderate	Low	Low	Low	Moderate	Moderate	Moderate

Note: ROBINS-I [31] assessed seven domains: D1, confounding; D2, selection of participants; D3, classification of interventions; D4, deviations from intended interventions; D5, missing data; D6, measurement of outcomes; and D7, selection of the reported result. Domain-level judgments were classified as low, moderate, serious, or critical risk of bias, or no information. Overall risk of bias was derived according to ROBINS-I guidance: low when all domains were judged low; moderate when all domains were low or moderate; serious when at least one domain was serious but none was critical; and critical when at least one domain was critical. "No information" was used when insufficient information precluded a definitive judgment.

Supplementary Table S4. Quantitative and contextual diagnostic data across included studies

Code	Age current	Age at diagnosis	Delay intervals	Definition of "delay"	Health care	Clinical structure	Setting (level)	Physician	Region	Referral pathways	Sex	SES	Parental educational level
1	Mean = 90.0 months (SD 49.6); range 36-204	NR	NR	NR	Mixed	NA	Community	Specialist (neurology/ psychiatry)	North, Northeast, Midwest, Southeast, South	NR	83.1% male, 16.9% female	Predominantly middle–high SES (insurance & education profile)	71.6% university or higher
2	NR	Mean 66.0 months (SD 34.8) Early diagnoses = 30.4%	Not calculated; binary early/late classification	Early <48 months; late ≥48 months (30.4% early)	Public (SUS)	Public	Specialty	Not specified	North, Northeast, Midwest, Southeast, South	PHC; spontaneous demand; emergency; CAPSi; day hospital; psychiatric hospital; PHC and spontaneous demand associated with early diagnosis	81.9% male; not associated with early diagnosis ($p > 0.20$)	Municipal HDI (very low to high); not significant in multiple models.	Not available
3	Mean 79.2 months (SD 46.8)	Mean 47.3 months (SD 30.5)	27.0 months (calculated)	Difference between age at first caregiver concern and diagnosis	Mixed	Mixed	Specialty	Generalist; specialist (neurology/ psychiatry)	Global (Brazil Included)	NR	83.6% male, 16.4% female, 0% other	NR	Elementary or less 1.1%; High school 1.4%; Associate 26.5%; Undergraduate 34.0%; Graduate 36.9%; None 0%
4	Mean 93.0 months (SD 48.4)	Mean 59.6 months (SD 40.5)	Mean 36 months	Time from first parental concern to formal diagnosis	Mixed	Public	Tertiary	Generalist	Southeast	First concern → pediatrician → no referral → eventual specialty diagnosis	89.5% male (17), 10.5% female (2)	Mostly middle-class	NR
5	26–50 months (70%)	NR	NR	NR	NR	Public	Community and specialty	NR	Southeast	Caregiver suspicion, information seeking, professional consultation; attribution/normalization (e.g., prematurity) before help seeking	95% male	Caregiver employment 10% formal	50% secondary

Code	Age current	Age at diagnosis	Delay intervals	Definition of "delay"	Health care	Clinical structure	Setting (level)	Physician	Region	Referral pathways	Sex	SES	Parental educational level
6	36-108 months	NR	NR	NR	Public (SUS)	Public	PHC and specialty	Generalist; specialist (neurology/ psychiatry)	Midwest	School concern → referral → specialized evaluation; travel to state capital; multiple consultations and tests	NR	NR	NR
7	NR	NR	NR	NR	Public (SUS)	Public	PHC and specialty	Generalist	Southeast	PHC suspicion → referral to CAPSi for assessment and care coordination	NR	NR	NR
8	Mean 120.5 months (SD 26.2)	Mean 79.2 months (SD 32.4)	49.3 months (calculated from reported means)	Interval from first parental concern to diagnosis	Public (SUS)	Public	Tertiary	Specialist (neurology/ psychiatry); Generalist	Southeast	Neurologists (most frequent); school staff; psychologists; pediatricians (1.9%)	97.3% male (36/37)	92.5% public school enrollment (proxy lower SES)	High school most common

Supplementary Table S5. Mapped barriers and missed opportunities in the ASD diagnostic pathway

Code	Barrier / Missed opportunity level	Barrier / Missed opportunity type	Gender-related?	Evidence type	Short excerpt (≤1–2 lines) or statistic	Key notes
1	Service, System, Family	Long waiting times for appointments, Financial constraints, Regional inequities in service availability, Absence of specialized services	No	Quantitative Association, Author Interpretation	**Barrier 1** - Long waiting times for appointments: "waiting lists: 59.6%"; **Barrier 2** - Financial Constraints: "cost of treatment as a barrier: 38.7%"; **Barrier 3** - Regional inequities in service delivery: "The number of institutions is unevenly distributed among the five Brazilian regions."; **Barrier 4** - Absence of specialized services: "Lack of services/treatment: 30.7%"	Waiting list barrier significantly associated with younger age (3–6 years), countryside location, public health use, and lower caregiver education; Lack of specialist services significantly associated with countryside location and lower caregiver education; Structural inequities across Brazilian regions
2	Provider, Service, System, Cultural/ Structural	Long waiting times for appointments, gender norms affecting symptom recognition, Regional inequities in service availability, Specialist shortages, Failure to apply screening methods, Delayed initiation of diagnostic evaluation	Yes	Author Interpretation, Quantitative Association	**Barrier 1** - Long Wait time for appointments: "Late diagnosis… may include pediatricians' non-adherence to evaluation protocols, variation in experience, and the use of non-validated instruments."; **Barrier 2** - Failure to apply screening tools: "Reasons may include pediatricians' non-adherence to evaluation protocols… and the use of non-validated instruments."; **Barrier 3** - Gender norms affecting symptom recognition: "Access barriers were more perceived by caregivers of female preschool children."; **Barrier 4** - Regional inequities in service availability: "Early diagnosis lower in North region; higher in Northeast and Southeast."; **Barrier 5** - Specialist shortages: "There is heterogeneity in the training and distribution of professionals in Brazil and the availability of multidisciplinary teams."; **Missed Opportunity 1** - Delayed initiation of diagnostic evaluation: "Only 30.4% diagnosed before 48 months"	Early diagnosis improved over time (2014–2018 increase), but remains low (≈30%); Regional disparities significant, North consistently later diagnosis. Highest proportion of N in Southeast (57.7%); Referral from PHC increases probability of early diagnosis; Gender associated with Barrier 3 only - gender norms affecting symptom recognition; Model explained only 2% of variance → strong contextual factors missing.
3	System	Overburdened public health system (SUS), Delayed confirmation of ASD diagnosis	No	Quantitative Association	**Barrier 1** - Overburdened public health system (SUS): "Public health coverage predicted later diagnosis (+3.22 months) in the regression model (Brazil included in analytic sample)." **Missed Opportunity 1** - Delayed confirmation of ASD diagnosis: "Brazil mean age at first concern = 20.3 months; mean age at diagnosis = 47.3 months → ~27-month gap."	Diagnosis in Brazil is predominantly made by specialists (neurologists 48.7%); PHC physicians involved in only 1.2% of first diagnoses; Public health coverage associated with later diagnosis (+3.22 months) in the regression model (Brazil included in analytic sample; effect not Brazil-only).

Code	Barrier / Missed opportunity level	Barrier / Missed opportunity type	Gender-related?	Evidence type	Short excerpt (≤1–2 lines) or statistic	Key notes
4	Provider, Service, System	Dismissal of caregiver concerns, Inadequate developmental surveillance, Absence of routine screening protocols, Delayed confirmation of ASD diagnosis	No	Quote, Quantitative Association, Author Interpretation	**Barrier 1** - Dismissal of caregiver concerns: "Most mothers described their interactions with the pediatrician as negative experiences (56.3%), as they felt discouraged to express their concerns again." AND "Pediatricians told mothers: “children should not be compared to each other,” and “boys have a slower development rate…”; **Barrier 2** - Inadequate developmental surveillance: "One-third (36.9%) of children first seen by a pediatrician were evaluated or referred for suspicion of atypical development."; **Barrier 3** - Absence of routine screening protocols: "In most Brazilian public health facilities, validated protocols or screening algorithms for early ASD detection have not been implemented."; **Missed Opportunity 1** - Delayed confirmation of ASD diagnosis: "Formal diagnosis occurred at mean age 59.6 months; mean delay = 36 months."	Diagnostic delay in Brazil (~36 months) mirrors LMIC patterns; High proportion of early parental concern (<24 months), but provider dismissal common; Clear provider-level missed opportunity at first pediatric contact.
5	Family, Provider	Normalization of symptoms ("wait and see"), "Wait and see" clinical approach, Inadequate developmental surveillance, Multiple consultations without ASD suspicion	No	Quote, Author Interpretation	**Barrier 1** - Normalization of symptoms ("wait and see"): caregiver reports family/doctor attributing delays to prematurity; **Barrier 2** - "Wait and see" clinical approach: same sequence includes clinician downplaying concerns; **Barrier 3** - Inadequate developmental surveillance: caregiver notes pediatrician observed delays/skill loss, implying late recognition after clear signs; **Missed Opportunity 1** - Multiple consultation without ASD suspicion: caregivers report prolonged interpretation as prematurity + only later, suggesting a period of contact/interpretation without ASD suspicion.	Qualitative caregiver-pathway content exists (“suspicions and search for information”; “feelings and paths” to confirm diagnosis); Strong caregiver gender skew (90% cis women) and young child sample (0–6); Provides mechanism-level evidence of normalization/prematurity explanations delaying help-seeking (qualitative).
6	Family, Provider, Service, System	Normalization of symptoms ("wait and see"), Transportation / geographic access Barriers, Low ASD knowledge or training, Fragmented care pathways, Specialist shortages, Long diagnostic queues	No	Quote, Author Interpretation	**Barrier 1** - Normalization of symptoms ("wait and see"): “...the mother watches… keeps a little quiet, thinking it may be a phase thing; **Barrier 2** - Transportation or geographic access barriers: “...I had to travel directly to the capital… had to leave three days of service...”; **Barrier 3** - Low ASD knowledge or training: “...it is necessary that the team is trained to recognize the first signs of ASD and carry out appropriate referral...”; **Barrier 4** - Fragmented care pathways: “...you go to five types of doctors or more… run after school paperwork, everything.”; **Barrier 5**: Specialist shortages: “...absence of specialized services… families went to the State capital to obtain assistance from specialists to obtain the diagnosis.”; **Barrier 6** - Long diagnostic queues: “...waiting time of some families for specialized services until the confirmation report by a specialist.”	School is repeatedly presented as a key trigger for identification and referral to evaluation; Geographic access to specialists (travel to capital) is a central obstacle in the diagnostic trajectory; Half the children had diagnostic hypothesis only; authors attribute this partly to waiting time for specialist confirmation.

Code	Barrier / Missed opportunity level	Barrier / Missed opportunity type	Gender-related?	Evidence type	Short excerpt (≤1–2 lines) or statistic	Key notes
7	Provider	Low ASD knowledge or training, Multiple consultations without ASD suspicion	No	Author Interpretation, Quantitative Association	**Barrier 1** - Low ASD knowledge or training: "many studies have shown that [pediatricians/general practitioners] are not trained to identify ASD." AND Knowledge improved after training (6.73 → 9.18; $p < 0.01$);<br><br>**Missed Opportunity 1** - Multiple consultations without ASD suspicion: "Even when parents suspect the condition and seek assistance, there is usually a delay until proper diagnosis and care are achieved."	Provider Characteristics: 68.2% female providers (15/22), Mean provider age: 46 years (SD 11.8), Mean time since graduation: 20.7 years (SD 12); Main findings: ASD knowledge score improved: mean 6.73 → 9.18 ($p < 0.01$); referrals of suspected ASD increased (4 months before training 11 referred, 1 suspected 4 months after training 13 referred, 6 suspected); Earlier diagnoses before training: "only two of the six… were aged over 4 years"; Demonstrates a feasible SUS-embedded intervention: brief training for PHC providers linked to CAPSi referral pathway.
8	Provider, Service, System, Cultural/ Structural	Low ASD knowledge or training, Inadequate developmental surveillance, Absence of routine screening protocols, Limited availability of screening tools, Specialist shortages, Social inequality, Delayed confirmation of ASD diagnosis	No	Author Interpretation, Quantitative Association	**Barrier 1** - Low ASD knowledge or training: "There are still health professionals without adequate training for the early detection of signs of ASD.";<br><br>**Barrier 2** - Inadequate developmental surveillance: "Although pediatricians are responsible for following up development… we observed a low rate of referral by these professionals.";<br><br>**Barrier 3** - Absence of routine screening protocols: "The use of screening tools is still limited in Brazil.";<br><br>**Barrier 4** - Limited availability of screening tools: "Challenges… include the high cost of training, limited access to tools, and lack of adequately qualified professionals.";<br><br>**Barrier 5** - Specialist shortages: "Low number of trained professionals and limited access contribute to late diagnosis.";<br><br>**Barrier 6** - Social Inequality: "Most participants were enrolled in public schools… individuals from economically disadvantaged backgrounds tend to have more difficulty accessing health care, potentially contributing to delayed diagnoses.";<br><br>**Missed Opportunity 1** - Delayed confirmation of ASD diagnosis: "Mean age at diagnosis was 79.2 months."	Late diagnosis (~79.2 months), longer than previous Brazilian study (~36 months); Pediatrician referral proportion very low; Prior interventions (speech therapy, psychotherapy) did not reduce diagnostic delay.

Note: Author interpretation refers to the interpretation or discussion provided by the authors of the original included study and is distinguished from empirical findings (quantitative associations), participant quotations, and review-level synthesis.

Supplementary Table S6. Comparative characteristics of reviews related to autism

Dimension	Matos 2022	Sukiennik 2022	Gomes 2015	Current Scoping Review (2026)	Comparative Interpretation
**Main Focus**	Age at diagnosis across income levels	Diagnostic tools & structural barriers in Brazil	Family burden & coping strategies	Diagnostic delay, screening gaps, missed opportunities, structural inequities	Extends previous reviews by integrating system-level missed opportunities
**Mean Age at Diagnosis**	~56 months; delay ~32 months	Reports high average age; ~3-year delay cited	Diagnostic postponement reported	Mean age at diagnosis varied across studies: 47.3–79.2 months; 66 months in the national CAPSi dataset; directly reported diagnostic delays ranged from approximately 24–36 months, with an additional calculated estimate of 49.3 months in one tertiary-care study.	Confirms persistent delay; identifies extreme tertiary delays
**Income / SES Effects**	No statistical difference between HIC vs LMIC	Wealth concentration & SUS inequalities described	Poor access to health services increases burden	Public health coverage associated with later diagnosis in multinational analysis including Brazil; SES proxies identified	Diverges from Matos (income-level comparison); confirms national inequities within Brazil
**Regional Inequities**	Not Brazil-specific	South/Southeast concentration of services	Studies mostly from South/Southeast	North associated with later diagnosis; specialist concentration in Southeast	Adds quantified regional effect (novel contribution)
**Provider Barriers**	Limited discussion	Low training; lack of tools; dismissal of concerns	Pilgrimage to hospitals and visits to countless healthcare professionals; diagnostic postponement	Inadequate surveillance; screening failure; dismissal; low primary-care involvement	Confirms and operationalizes provider-level mechanisms

**Dimension**	**Matos 2022**	**Sukiennik 2022**	**Gomes 2015**	**Current Scoping Review (2026)**	**Comparative Interpretation**
**Screening Tools**	Need for standardized early tools	Limited translation/validation; scarce implementation	Screening discussed but not central	Absence of routine screening protocols; failure to apply screening methods	Moves from "tool availability" to "implementation failure"
**Primary Health Care Role**	Not deeply analyzed	Training improves referrals (Bordini 2015)	Parents discouraged by pediatricians	PHC rarely serves as the first diagnostic level; referral from PHC improves early diagnosis	Confirms training effect; highlights structural gap in PHC
**Gender Differences**	Boys diagnosed more often	Notes male predominance	Not focus	Caregivers of girls report more barriers	Novel gender-access insight (limited evidence)
**Structural/System Barriers**	Highlights global delay	Wealth inequality; specialist shortage	Poor access & fragmented care	Long waiting lists; fragmented pathways; travel to capitals; specialist shortages	Strong confirmation and expanded system mapping
**Caregiver Experience**	Recognizes parental delay	Doctor–parent relationship issues	Emotional overload; stress; diagnostic pilgrimage	Normalization ("wait and see"); multiple consultations without suspicion	Confirms psychosocial mechanism; links to missed screening
**Methodological Scope**	Global systematic review	Brazilian systematic review	Brazilian family-centered systematic review	Multi-level scoping review (family, provider, system, structural, policy)	Methodological novelty: multi-level mapping framework
**Policy Implications**	Need for standardized diagnosis	Need for training & tool expansion	Need for family support & SUS strengthening	Strengthening developmental surveillance and early identification; regional resource redistribution; PHC capacity building	Expands toward governance and implementation science
